# Vocal behaviour predicts mating success in giant pandas

**DOI:** 10.1098/rsos.181323

**Published:** 2018-10-31

**Authors:** Benjamin D. Charlton, Meghan S. Martin-Wintle, Megan A. Owen, Hemin Zhang, Ronald R. Swaisgood

**Affiliations:** 1 San Diego Zoo's Institute for Conservation Research, California, CA 92027-7000, USA; 2 PDXWildlife, 9233 SW Brier Pl, Portland, OR 97219, USA; 3 China Research and Conservation Centre for the Giant Panda, Sichuan province, People's Republic of China

**Keywords:** vocal communication, giant pandas, mating behaviour

## Abstract

Surprisingly little is known about how mammal vocal signals are used to achieve behavioural synchrony in the lead up to copulation. The ability to signal short-term fluctuations in arousal levels and behavioural intention is likely to be particularly important for synchronizing mating behaviour in asocial species, which must overcome their natural avoidance and aggressive tendencies to mate. Here, we examined vocal behaviour during breeding encounters in captive giant pandas (*Ailuropoda melanoleuca*) to gain a greater understanding of how close-range vocal signalling mediates reproduction in this asocial, and conservation-dependent species. Our results revealed that the occurrence of different giant panda vocalizations and acoustic variation within these calls is predictive of successful encounters leading to copulation, as opposed to unsuccessful encounters that do not. In addition, key differences were detected between vocalizations produced during and just prior to copulation. These findings illustrate that vocal exchanges are crucial for achieving behavioural synchrony and signalling intention to mate in giant pandas, and could also provide a valuable tool for breeding programmes, helping conservation managers to assess the likelihood of breeding introductions leading to copulation or potentially injurious failure.

## 1. Introduction

Although several studies have confirmed that mammal vocal signals conveying some aspect of male quality are used to attract mates [1,2], far less is known about how mammal vocalizations are used to synchronize mating behaviour once close contact between a male and female has been made. During close encounters, acoustic cues to the caller's physical attributes (such as size, age, sex or hormonal state) are likely to be important (for a review see [3]); however, ‘dynamic’ acoustic variation within calls signalling short-term fluctuations in arousal levels and behavioural intention may also assume critical importance for asocial species that need to overcome their natural avoidance and aggressive tendencies to mate. In this study, we examined vocal behaviour during close-range intersexual interactions in an asocial mammal, the giant panda (*Ailuropoda melanoleuca*), to gain a greater understanding of how vocal signalling mediates reproduction in this endangered species.

Giant pandas are obligate bamboo foragers that inhabit the remote, mountainous regions of central China [4]. Relatively solitary by nature, free-ranging giant pandas occupy distinct seasonal ranges [5] and typically avoid other conspecifics outside of the annual breeding season [4]. Effective communication is, therefore, crucial for male and female giant pandas not only to locate opposite-sexed individuals for mating purposes, but also to overcome their natural avoidance and aggressive tendencies in order to negotiate the delicate courtship process leading to mating once close contact is established [6,7]. Olfaction is undoubtedly a key sensory modality in this species' sexual communication [8]; however, the occurrence of vocalizations in both sexes also increases significantly during the breeding season [6,7,9,10], indicating that vocal signals are important for coordinating reproduction [4]. The giant panda's most conspicuous vocalization is a bleat that is thought to signal non-aggressive intent and promote contact between individuals [10–12]. Female giant pandas also produce high-pitched tonal vocalizations called chirps almost exclusively during their oestrus period [7,11]. While chirping is relatively uncommon in males, both sexes also produce barks, moans, roars, growls and squeals when they interact with other conspecifics, and honks predominantly when they are alone (for a description of these calls see [12]). Although the function of honks is unknown, it is generally assumed that barks, growls and roars are aggressive calls that are produced during agonistic encounters, squeals are produced by subordinate individuals during or after a fight, and moans denote mild aggression or ambivalent motivation [10–12].

More recent studies have used a source-filter theory [13] approach to describe the acoustic structure of giant panda vocal signals. The source-filter theory states that vocal signal production is generated by the conversion of airflow from the lungs to acoustic energy by the larynx, the source, which is subsequently filtered by the vocal tract. The source signal determines the fundamental frequency (F0) of the vocalization and the supra-laryngeal vocal tract acts as a spectral filter, selectively amplifying certain frequencies termed vocal tract resonances or ‘formants’ [14]. Applying this approach to giant panda bleats has revealed that the rate of ‘vibrato-like’ F0 modulation in male bleats is positively correlated to male androgen levels [15], mean F0 and formants encode information about the caller's identity [16], and the formant spacing in bleats varies according to the sex and size of callers [17]. Subsequent playback studies have also demonstrated that giant pandas attend to this potentially important information during the breeding season [18–20]. Additional studies have shown that male giant pandas perceive acoustic differences in female chirps that would allow them to maximize the chances of conception [21], and produce longer duration bleats with higher mean F0 during vocal interactions with peak oestrous females [22], indicating that bleat duration and F0 are reliable cues to the caller's motivational state in this species.

The observation that males bleat at high rates during the breeding season, and especially when they interact with females [10,11] or encounter female odours [23], indicates that this vocalization is likely to signal important information about the vocalizer's size, identity, sex, hormonal quality and motivational state in reproductive contexts. Acoustic variation within other call types may also be important for giant pandas to signal rapid changes of mood to conspecifics and achieve behavioural synchrony just prior to and during copulation. Mammal vocalizations given in high arousal contexts are often characterized by increased duration and F0 [24–27]. Accordingly, increased duration and F0 in giant panda vocalizations could signal high sexual motivational and subsequent willingness to mate. Vocal signals delivered by highly aroused giant pandas may also contain more nonlinear phenomena, due to irregular or chaotic vocal fold vibration patterns caused by high sub-glottal air pressures (for a discussion of nonlinear phenomena, see [28–30]). Furthermore, because male and female giant pandas can be aggressive towards one another during mating attempts [31], the occurrence of certain vocal signals and/or characteristics could also signify whether a breeding encounter is moving towards copulation or a potentially serious injury to one or both of the animals, which would not only refine our understanding of the functional relevance of different giant panda call types, but also allow observations of vocal behaviour to be used to promote successful copulation while ensuring the safety of the animals [32].

The primary aim of the current study was to investigate whether call usage and acoustic variation within giant panda vocal signals during breeding introductions is predictive of successful encounters leading to copulation, as opposed to unsuccessful encounters that do not. For successful breeding introductions, we also examined differences in the acoustic structure of vocal signals delivered prior to and during copulation. We predicted that giant pandas would produce more bleats and chirps, and fewer aggressive vocalizations, such as roars and barks, during the pre-copulatory phase of successful breeding introductions than during unsuccessful breeding introductions. In addition, we predicted that the pre-copulatory phase of successful breeding introductions would be characterized by long duration male bleats with increased F0 modulation, which may be expected to promote contact and maximally highlight male hormonal quality [15]. We also predicted that vocalizations produced during copulation would be characterized by increased duration and mean F0, and a greater prevalence of nonlinear phenomena than those produced prior to copulation taking place, due to the heightened arousal state associated with this context [25,33,34]. We had no strong *a priori* prediction for the effect of formant spacing on breeding outcome. Our findings will improve knowledge of this endangered species' sexual communication, and could also help conservation managers to assess the likelihood of breeding introductions leading to copulation, and in doing so provide a valuable tool for captive breeding programmes.

## 2. Material and methods

### 2.1. Study site and animals

The giant pandas involved in this study were housed at the China Research and Conservation Centre for the Giant Panda, at Bifengxia near Ya'An, Sichuan, China and Shenshuping in Gengda, Sichuan, China. Behavioural and acoustic data were collected from 23 adult giant pandas (eight males and 15 females) during the 2016 and 2018 breeding seasons (February–May). All the animals had previous mating experience prior to the study, were sexually mature (ages ranging from 6 to 18 years) and individually recognizable (housing and animal husbandry practices are described in [35,36]). During breeding introductions, giant pandas were housed in concrete-walled, open-air enclosures (6 × 15 m) with an indoor enclosure area (3 × 8 m). All enclosures had one barred ‘howdy’ window and a circular barred gate located on the long sides of the enclosure (four potential interaction windows, two per side). Enclosures were arranged in a large circle so giant pandas could be moved freely between pens for mate pairings. In this configuration, giant pandas shared walls with two other animals, except for animals residing in the end enclosures, which only had one neighbour. Giant pandas were exposed to natural light conditions and were fed a diet of local bamboo supplemented with bread, high-fibre biscuits, carrots and apples. Animal care and use guidelines of the American Society of Mammalogists [37] were followed by all facility operators.

### 2.2. Recordings

**Figure 1.**
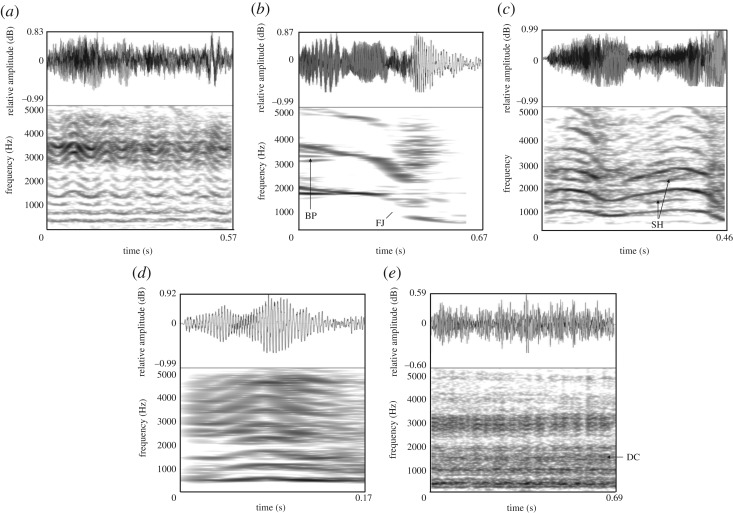
Waveforms and spectrograms of the giant panda vocalizations produced during breeding introductions. Spectrogram settings: fast Fourier transform (FFT) method; window length = 0.03 s; time step = 0.002; frequency step = 20 Hz; Gaussian window shape; dynamic range = 40 dB. Bleats (*a*), chirps (*b*), moans (*c*), barks (*d*) and roars (*e*) were separately clustered as discrete call-types. Nonlinear phenomena are signified as follows: DC, deterministic chaos; FJ, frequency jump; BP, biphonation; SH, subharmonics.

Vocalizations were recorded during 21 breeding introductions (when a female is placed in the male's enclosure for breeding purposes) using RØDE NTG-2 directional microphones attached to Zoom H4N portable solid-state digital recorders (Tokyo, Japan; sampling rate 44.1 kHz, amplitude resolution 16 bits) at distances ranging from 2 to 15 m. The recordings were then normalized to 100% peak amplitude, converted to mono, and saved as WAV files (44.1 kHz sampling rate and 16 bits amplitude resolution). We noted the identity of the vocalizing animal on the recordings in order to retrospectively assign vocalizations to individuals, and grouped recording sequences according to whether the breeding introductions were successful (*N* = 13) or unsuccessful (*N* = 8) (refer to ‘Mating procedure & reproductive performance’ for details). Vocalizations produced during successful introductions were further partitioned into those given during the pre-copulatory phase (before male intromission) and those given during copulation. The overall spectral structure of each call was initially investigated in Praat v. 5.1 (www.praat.org) using narrow-band spectrograms (FFT method, window length = 0.03 s, time steps = 250, frequency steps = 1000, Gaussian window shape, dynamic range = 40 dB) and recordings were categorized as bleats, chirps, moans, barks and roars based on qualitative descriptions of these calls [12] (figure 1). Honks, squeals and growls were not observed in any of the breeding introductions.

### 2.3. Acoustic analysis

The acoustic analyses were performed on 2566 vocalizations (757 male bleats, 230 female bleats, 908 female chirps, 465 female moans, 145 female barks and 61 female roars) given during breeding introductions using custom-built programmes in Praat 5.3.85 DSP package [38] that automatically extract and measure a range of acoustic measures. The outputs were checked against the corresponding spectrograms to ensure that Praat accurately tracked and measured all acoustic features. In addition, we documented whether nonlinear phenomena were observed in the spectrograms. Nonlinear phenomena are visualized as additional spectral components called *subharmonics* that appear at integer fractional values of an identifiable F0, (e.g. F0/2, F0/3 etc.), abrupt discontinuous changes in F0 called *frequency jumps*, episodes of non-random noise termed *deterministic chaos* and the occurrence of two simultaneous but independent fundamental frequencies, termed *biphonation*. The acoustic data are provided as electronic supplementary material (Dataset.sav).

#### 2.3.1. Analysis of male and female bleats

The duration of individual bleats was measured directly from the waveform and the F0 contour was extracted using the ‘To Pitch (cc)’ command in Praat, with a search range of 200–850 Hz and a time step of 0.01 s. Time-varying numerical representations of the F0 contour were then checked for any incorrect values (using the ‘Edit’ window in Praat) before the mean F0 value (mean F0) was measured. In addition, to quantify the characteristic F0 modulation of giant panda bleats, we measured the number of complete cycles of F0 modulation per second (F0 modrate) and the average peak-to-peak variation of each F0 modulation (F0 modextent).

The frequency values of the first six formants of bleats were measured using Linear Predictive Coding (LPC; ‘To Formants (Burg)’ command) and the following analysis parameters: time step: 0.01 s; window analysis: 0.2 s; maximum formant value: 3800–4000 Hz; maximum number of formants: 6; pre-emphasis: 50 Hz (for an overview of LPC see [39]). To more accurately measure the lower three formants, we ran a second analysis in which the maximum formant value was changed to 2000 Hz and the maximum number of formants to three. The formant values from both analyses were then combined (F4, F5, F6 from the first analysis and F1, F2, F3 from the second analysis) and used to estimate the formant spacing (ΔF) during each bleat using the linear regression method [40]. The linear regression method requires the expected formant positions to be plotted against actual measured values, and this in turn requires a vocal tract model to provide the expected formant values to regress the observed values against. Because giant panda bleats are delivered with a partially or fully closed mouth [12] and do not appear to be fully nasalized, we modelled the vocal tract as a tube closed at both ends (*sensu* [16]).

#### 2.3.2. Analysis of female chirps, moans and barks

Formants are not observed in female giant panda chirps due to the relatively high F0 and the concomitant decreased harmonic density that fails to adequately sample these spectral components [35]. Spectral peaks that are likely to represent formants could also not be consistently measured in moans or barks. As a result, the analysis of these calls focused on F0 characteristics. To measure F0 parameters, we extracted the F0 contour using the ‘To pitch (cc) command’ (time step = 0.01 s; minimum and maximum F0 = 100 Hz and 2500 Hz, respectively). Time-varying numerical representations of the F0 contour were then compared with the F0 contour as visualized on a spectrogram and checked to see if Praat was correctly tracking the F0. In cases where Praat incorrectly tracked a harmonic (or sub-harmonic), numerical representations of the F0 contour were adjusted using the ‘Edit’ window in Praat before mean F0 was measured. The duration of each call was taken directly from the waveform and narrow-band spectrograms (FFT method, window length = 0.03 s, time steps = 250, frequency steps = 1000, Gaussian window shape, dynamic range = 40 dB) were used to determine the presence of nonlinear phenomena.

#### 2.3.3. Analysis of female roars

F0-related features were not extracted from giant panda roars because they consist of broadband frequency noise without an observable F0. However, distinct energy bands were observed within the broadband frequency noise of these calls that are likely to represent formants. Roars are produced with an open mouth [12]. Therefore, to measure these spectral peaks (hereafter referred to as formants), we used Linear Predictive Coding (LPC; ‘To Formants (Burg)’ command, time step, 0.01 s; window analysis, 0.05 s, pre-emphasis, 50 Hz), and modelled the giant panda vocal tract as a tube open at one end (the mouth) and closed at the other (the glottis). Previous work revealed that the giant panda's vocal tract length is around 32 cm [16]. A 32 cm linear tube open at one end and closed at the other acts as a quarter-wave resonator, which should produce a first formant frequency (F1) at approximately 273 Hz according to the formula c/4 L (in which L = length and c is the speed of sound: 350 m per second in the moist air of the vocal tract), and subsequent formants at F1*3, F1*5, F1*7, etc. (for more details see [16]). Prior to the formant analysis, visual inspection of the spectral acoustic structure of roars also allowed us to confirm that four prominent frequency components exist below 2200 Hz that are likely to represent formants. Accordingly, we set the analysis parameters to search for four formants below 2200 Hz. The formant values were then used to estimate the formant spacing (ΔF) during each call using the linear regression method of Reby & McComb [40] with a ‘open-one-end’ vocal tract model. The duration of each roar was taken directly from the waveform and narrow-band spectrograms (FFT method, window length = 0.03 s, time steps = 250, frequency steps = 1000, Gaussian window shape, dynamic range = 40 dB) were used to determine the presence of nonlinear phenomena.

### 2.4. Mating procedure and reproductive performance

**Figure 2.**
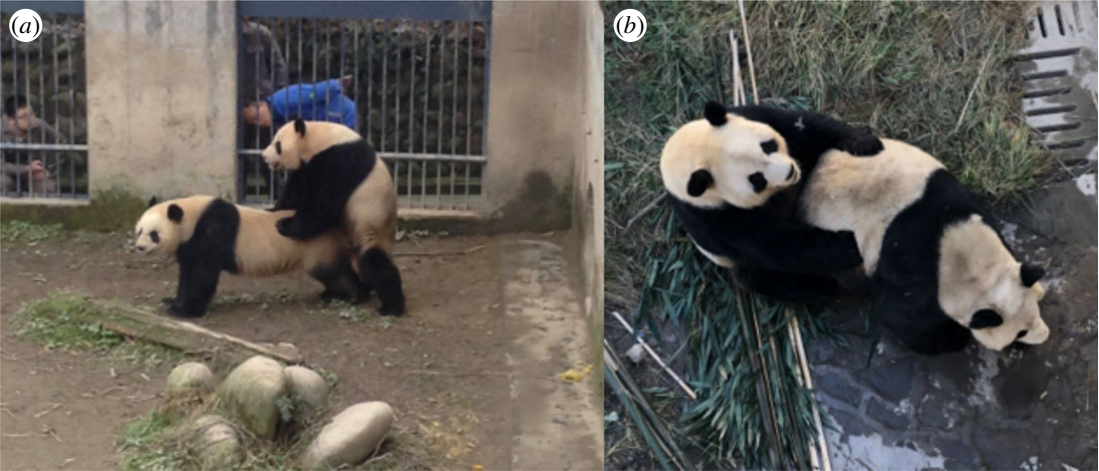
A successful breeding introduction. A breeding introduction is deemed to be successful once the male achieves intromission. Successful copulation is shown in the standing position (*a*) and the ‘roll-back’ position (*b*).

Female giant pandas in peak oestrous (verified using hormonal assays: [36]) were selected for breeding introductions during the breeding season (February to June), and subsequently paired with a genetically compatible partner as determined by inbreeding coefficients obtained from pedigree analysis. Mating was first attempted with the priority male according to the genetic management plan even if animals appeared indifferent toward the potential mate. Males were introduced to female pens for mating between 9.00 and 16.00. If physical aggression between male and female was observed, such as biting or lunging, then the animals were immediately separated. Breeding introductions were deemed to be ‘successful’ if the male achieved intromission (i.e. copulation occurs) (figure 2). In the current analysis, we therefore defined ‘breeding outcome’ as successful when copulation occurred with intromission, or unsuccessful when a breeding introduction did not lead to copulation (for more details about the mating procedure, see [41]).

### 2.5. Statistical analysis

The statistical analyses were conducted using IBM SPSS Statistics v. 20, significance levels were set at 0.05, and two-tailed probability values are quoted. First, we performed a two-step cluster analysis to confirm the provisional classification of giant panda vocalizations into five different call-types (bleats, chirps, moans, barks and roars) based on visual inspections of spectrograms and previous descriptions of call characteristics [12]. Two-step clustering was necessary due to the combination of binary (dichotomous) and continuous acoustic measures. A two-step cluster analysis partitions the data into a predefined number of relatively homogeneous groupings in order to minimize variability within clusters and maximize variability between clusters. To ensure that the variables for the cluster analysis were independent, we input the acoustic measures into a principal components analysis with varimax rotation, in order to reduce these variables into one latent variable with an Eigenvalue of 8.88 that explained 74.0% of the variance. This latent variable was saved using the ‘Anderson-Rubin’ method and entered into the two-step cluster analyses. To determine the best solution, with the highest silhouette measure, we used the log-likelihood distance measure and left the maximum number of clusters at 15 (the SPSS default).

Generalized linear mixed effect models (GLMMs) fitted with maximum-likelihood estimation were used to examine acoustic variation during breeding introductions. To determine whether call usage is predictive of breeding outcome, we compared the occurrence of different vocalizations produced during the pre-copulatory phase of successful breeding introductions (i.e. before male intromission) with those produced during unsuccessful breeding introductions that do not result in copulation. For this GLMM analysis, we entered call type (male bleat, female bleat, female chirp, female moan, female bark and female roar) as a fixed factor categorical independent variable and breeding outcome (successful or unsuccessful) as a binary logistic dependent variable. For the analysis of acoustic variation within calls produced prior to copulation in successful breeding introductions versus those produced during unsuccessful breeding introductions, separate models were run in which the acoustic measures for a given call type were entered as continuous independent predictor variables, the presence of nonlinear phenomena (yes or no) was entered as a categorical fixed factor, and breeding outcome (successful or unsuccessful) as a binary logistic dependent variable. To determine whether the acoustic structure of giant panda vocalizations produced during successful breeding introductions varied pre- and post-intromission, we entered copulation stage (pre-copulation or copulation) as a binary logistic dependent variable and the acoustic measures for each call type as predictor variables. Because we analysed multiple recordings from each subject, and in order to avoid temporal pseudoreplication, we entered subject identity as a random factor in all of the GLMMs. The direction of effects was determined using the slope of the coefficient estimates, and robust estimation of fixed effects and coefficients was used to handle any violations of model assumptions.

## 3. Results

### 3.1. Classification of giant panda vocalizations

Visual examination of giant panda vocalizations using narrow-band spectrograms resulted in five provisional call types: bleats, chirps, moans, barks and roars (figure 1). The two-step cluster analysis solution with the highest silhouette measure (of 9.0) was obtained using five clusters that corresponded to our original subjective classification of call types in 100% of cases. The use of a cluster analysis to objectively group vocalizations into five discrete groups confirms that these calls are acoustically distinct, and are subsequently referred to as bleats, chirps, moans, roars and barks. Descriptive statistics for the acoustic features of giant panda vocalizations measured in this study are given in table 1.

**Table 1.** Descriptive statistics for each of the acoustic measures. See text for definition of variables.

	male bleats	female bleats	female chirps	female moans	female barks	female roars
acoustic measures	*M* ± s.d.	*M* ± s.d.	*M* ± s.d.	*M* ± s.d.	*M* ± s.d.	*M* ± s.d.
duration (s)	0.9 ± 0.4	1.1 ± 0.6	0.3 ± 0.1	0.7 ± 0.4	0.2 ± 0.0	0.7 ± 0.3
mean F0 (Hz)	380.3 ± 61.0	384.1 ± 59.1	907.5 ± 355.7	421.0 ± 152.0	466.1 ± 111.6	—
F0 modrate (Hz)	11.4 ± 6.9	10.2 ± 6.7	—	—	—	—
F0 modextent (Hz)	96.8 ± 43.2	85.1 ± 39.5	—	—	—	—
nonlinear phenomena (% of calls)	0.0		41.3	13.2	30.0	100.0
deterministic Chaos (% of calls)	0.0		0.0	11.3	30.0	100.0
subharmonics (% of calls)	0.0		3.1	1.7	0.0	0.0
biphonation (% of calls)	0.0		0.2	0.2	0.0	0.0
frequency jumps (% of calls)	0.0		38.0	0.0	0.0	0.0
F1 (Hz)	467.2 ± 94.1	451.0 ± 81.4	—	—	—	445.8 ± 69.8
F2 (Hz)	1052.2 ± 180.1	1031.0 ± 184.7	—	—	—	963.8 ± 79.5
F3 (Hz)	1667.2 ± 308.8	1629.6 ± 257.0	—	—	—	1467.4 ± 151.8
F4 (Hz)	2427.7 ± 266.9	2367.8 ± 206.6	—	—	—	1901.6 ± 78.7
F5 (Hz)	3023.9 ± 211.8	3012.7 ± 179.8	—	—	—	—
F6 (Hz)	3398.2 ± 168.9	3425.4 ± 99.4	—	—	—	—
ΔF (Hz)	580.1 ± 36.5	576.8 ± 26.4	—	—	—	571.1 ± 26.7

### 3.2. Does the occurrence of different call types predict breeding outcome?

**Figure 3.**
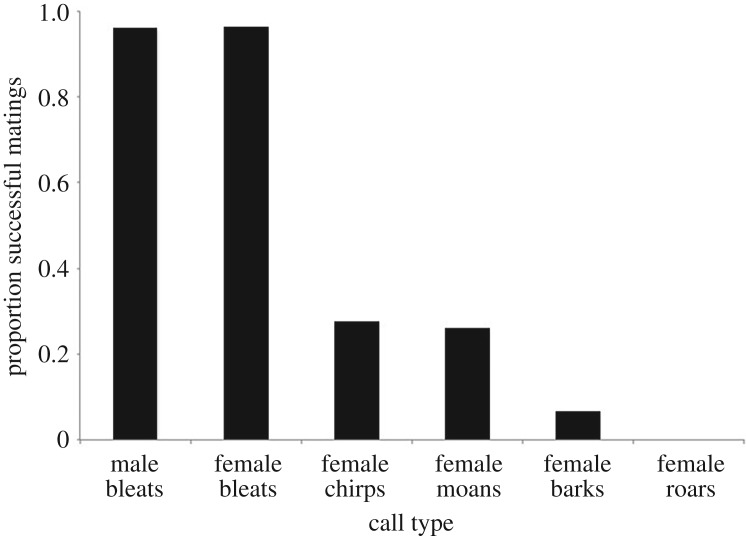
Proportion of different giant panda vocalizations produced during the pre-copulatory phase of successful breeding introductions. A GLMM analysis was used to compare the occurrence of different vocalizations produced during the pre-copulatory phase of successful breeding introductions with those produced during unsuccessful breeding introductions that did not result in copulation. For the analysis, breeding outcome (successful = 1 or unsuccessful = 0) was entered as a binary logistic dependent variable, call type as a fixed factor categorical independent variable, and subject identity as a random factor. Estimates closer to 1 indicate that a given call is more predictive of a successful breeding introduction leading to copulation, and vice versa.

Call usage varied significantly according to breeding outcome (*F*_5,1946_ = 654.47, *p* < 0.01). The pre-copulatory phase of successful breeding introductions was characterized by male and female bleats, confirming that these calls are highly predictive of successful breeding outcomes (figure 3). Female moans were also more strongly associated with successful than unsuccessful breeding outcomes (figure 3). In contrast, female roars were not observed during the pre-copulatory phase of successful breeding introductions (figure 3), making this call type 100% associated with breeding failure. Female barks and chirps were also more strongly associated with unsuccessful breeding outcomes (figure 3).

### 3.3. Does acoustic variation within giant panda vocalizations predict breeding outcome?

#### 3.3.1. Male and female bleats

We found that male bleats produced during the pre-copulatory phase of successful breeding introductions were longer in duration than those produced during unsuccessful breeding introductions (table 2). Breeding outcome was not significantly related to the mean F0, F0 modextent, F0 modrate or ΔF of male bleats (table 2). Female bleats given during the pre-copulatory phase of successful breeding introductions had lower F0 modextent and higher ΔF than those produced during unsuccessful breeding introductions (table 2). No other acoustic feature of female bleats differed between the recording conditions (table 2). Nonlinear phenomena were not observed in bleats.

**Table 2.** Relationships between breeding outcome and acoustic features of giant panda vocalizations. Significant correlations are highlighted in bold. ‘*e’* = coefficient estimate: positive coefficient estimates (*e*) denote acoustic measures were higher during successful breeding introductions and vice versa.

	male bleats			female bleats			female chirps			female moans			female barks		
acoustic measures	*e*	*F*_1,749_	*P*	*e*	*F*_1,226_	*P*	*e*	*F*_1,904_	*P*	*e*	*F*_1,461_	*P*	*e*	*F*_1,141_	*P*
duration (s)	0.53	4.04	**0.04**	4.37	1.99	0.16	0.61	3.42	0.07	0.73	0.96	0.33	−6.19	29.15	**<0.01**
mean F0 (Hz)	−0.00	0.58	0.45	−0.00	0.07	0.80	−0.01	190.09	**<0.01**	0.01	0.09	0.77	0.01	56.70	**<0.01**
F0 modextent (Hz)	0.02	2.59	0.11	−0.02	14.45	**<0.01**		—	—	—	—	—	—	—	—
F0 modrate (Hz)	0.04	0.44	0.51	0.01	0.00	0.96		—	—	—	—	—	—	—	—
nonlinear phenomena	—	—	—	—	—	—	−1.05	256.84	**<0.01**	−3.19	25.64	**<0.01**	−0.46	200.25	**<0.01**
ΔF (Hz)	−0.00	0.08	0.78	0.02	5.34	**0.02**		—	—	—	—	—	—	—	—

#### 3.3.2. Female chirps, moans, barks and roars

Female giant pandas produced chirps with lower mean F0 and less nonlinear phenomena during the pre-copulatory phase of successful breeding introductions than they did during unsuccessful breeding introductions (table 2). Female barks were shorter in duration, had higher mean F0, and less nonlinear phenomena during the pre-copulatory phase of successful breeding introductions (table 2). Moans produced during the pre-copulatory phase of successful breeding introductions also had less nonlinear phenomena than those produced during unsuccessful breeding introductions (table 2). The mean F0 and duration of female moans did not have statistically significant effects on breeding outcome (table 2). Female roars were not produced during the pre-copulatory phase of successful breeding introductions.

### 3.4. Does the acoustic structure of giant panda vocalizations differ according to copulation stage?

#### 3.4.1. Male and female bleats

Males produced bleats with higher mean F0 during copulation than they did during the pre-copulatory phase of successful breeding introductions (table 3). No other acoustic differences in male bleats were detected across the two recording conditions (table 3). Female bleats that were produced during copulation were shorter in duration, had higher mean F0 and ΔF and lower F0 modext than those produced during the pre-copulatory phase of successful breeding introductions (table 3). The F0 modrate of female bleats did not differ according to copulation stage (table 3).

**Table 3.** Relationships between copulation stage and acoustic features of giant panda vocalizations. Significant correlations are highlighted in bold. ‘*e’* = coefficient estimate: positive coefficient estimates (*e*) denote acoustic measures were higher during copulation than the pre-copulatory stage of successful breeding introductions.

	male bleats			female bleats			female chirps			female moans			female barks		
acoustic measures	*E*	*F*_1,541_	*P*	*e*	*F*_1,200_	*P*	*e*	*F*_1,566_	*P*	*e*	*F*_1,295_	*P*	*E*	*F*_1,56_	*P*
duration (s)	−1.24	1.83	0.18	−0.78	8.41	**<0.01**	−9.75	1.43	0.23	1.45	2.96	0.09	31.12	5.61	**0.02**
mean F0 (Hz)	0.01	4.68	**0.03**	0.02	32.97	**<0.01**	−0.00	0.68	0.41	−0.01	5.68	**0.02**	0.01	0.71	0.40
F0 modextent (Hz)	−0.00	0.05	0.82	−0.03	5.37	**0.02**	—	—	—	—	—	—	—	—	—
F0 modrate (Hz)	−0.08	1.61	0.21	−0.06	1.51	0.22	—	—	—	—	—	—	—	—	—
nonlinear phenomena	—	—	—	—	—	—	1.80	121.50	**<0.01**	−0.29	19.10	0.72	−1.33	5.35	**0.02**
ΔF (Hz)	0.00	0.52	0.47	0.02	14.35	**<0.01**	—	—	—	—	—	—	—	—	—

#### 3.4.2. Female chirps, moans, barks and roars

Female chirps that were produced during copulation had more nonlinear phenomena than those given in the lead up to copulation (table 3). Chirp duration and mean F0 did not significantly differ according to copulation stage (table 3). In addition, female barks produced during copulation were longer in duration and had less nonlinear phenomena than those produced prior to copulation (table 3). Bark mean F0 did not significantly differ across the two recording contexts (table 3). Moans produced during copulation had lower mean F0 than those produced during the pre-copulatory phase (table 3); however, the duration and prevalence of nonlinear phenomena in female moans did not differ according to copulation stage (table 3). Because female roars were only produced during copulation, the acoustic structure of these calls could not be compared across copulation stages.

## 4. Discussion

Our results demonstrate that the occurrence of specific giant panda vocal signals and acoustic variation within these calls during close-range intersexual interactions are predictive of successful copulation. Recent studies of this species' mate choice behaviour have revealed that giant pandas display adaptive mutual mate choice [41] and that certain personality traits are more compatible in mating contexts [42]. By using recordings captured during breeding introductions, the findings of the current study show unequivocally that vocal signals are also important for synchronizing this species’ mating behaviour. Based on the assumption that our findings generalize to free-ranging animals, the results of the current study represent the first systematic contribution to understanding how giant pandas employ vocal signals during the copulatory phase of mating.

In line with our predictions, the context-related differences in call usage revealed that considerably more bleats were produced during the pre-copulatory phase of successful breeding introductions when compared to unsuccessful introductions. These findings confirm that bleats are nonaggressive calls that function to promote contact between male and female conspecifics during the breeding season [12]. In addition, our analyses revealed significant acoustic variation within male and female bleats that is likely to be functionally relevant. As predicted, male bleats produced during the pre-copulatory phase of successful breeding introductions were longer in duration than those produced during unsuccessful introductions. Longer duration bleats may help to encourage close contact by reassuring females of nonaggressive intent, and could even promote female lordosis by acting as a behavioural releaser (e.g. chemical signals in boars: [43]). Female bleats were also significantly longer in duration during the pre-copulatory phase than during copulation, which further emphasizes that increasing bleat duration is likely to be important for promoting close contact.

Contrary to our *a priori* predictions, males did not produce bleats with significantly higher F0 modextent during successful breeding introductions. Instead, we found that females produced bleats with lower F0 modextent during the pre-copulatory phase of successful breeding encounters, and lower still during copulation, indicating that females modulate F0 over a narrower range of frequencies in these contexts. A possible reason for this may be found if we consider that F0 modulation extent is significantly lower in female giant panda bleats than it is in male bleats [17]. The production of bleats with lower F0 modulation extent could, therefore, help to assure males of the caller's female status, thereby limiting any potential male aggression and promoting copulation.

Interestingly, females also produced bleats characterized by higher formant spacing during the pre-copulatory phase of successful breeding introductions than they did during unsuccessful breeding introductions, and significantly higher formant spacing during the copulation phase of a successful breeding introduction. Formant spacing in giant panda bleats is a reliable cue to the caller's sex and male body size [17], but not significantly correlated with female body size. This is assumed to reflect the lack of a close relationship between skull and body size in female giant pandas [17], which then reduces the potential for formants to reliably signal body size. Another potential reason why formant spacing is not tightly correlated to female body size is that females make small ritualized adjustments of this acoustic parameter during the breeding season, in order to sound smaller and less threatening during close range interactions with males (the frequency coding hypothesis: [44]). Indeed, male giant pandas are quicker to respond and show more attention toward playback speakers broadcasting bleats with increased formant spacing mimicking smaller, and presumably less threatening callers [19]. The most plausible explanation is that bleats with higher formants receive greater attention from male giant pandas simply because they sound more ‘female-like’. It is conceivable, therefore, that females raise formants in bleats to sound less threatening and further reassure males of their female status.

It is not clear how female giant pandas would temporarily shorten their supra-laryngeal vocal tract length, however, because they do not appear to make facial adjustments during call production (e.g. the lip rounding and retraction seen in primates [45]) and are unlikely to be able to move the larynx further towards the oral cavity from its already high position (B Charlton 2009, unpublished data). One possible way to raise formants and decrease the acoustic impression of body size is to change the resonance characteristics of the vocal tract by adjusting the degree of mouth opening during call production. Giant panda bleats are certainly not produced with a wide-open mouth [12], but it is not always fully closed during bleat production, and this could have a subtle but perceptible effect on the formant spacing. If females are more likely to fully close the mouth during bleat production when they interact with males, then this would make the vocal tract behave more like a ½ wave resonator, thereby shifting formants upwards (for discussion of this, see [14]). The use of a closed mouth during bleat production may even have evolved for this precise function, i.e. to sound less threatening by raising formants. This intriguing possibility certainly merits further investigation. One possible way to test this hypothesis would be to use a sound visualization technique (an acoustic camera: [46]) to determine whether sound emission ceases to radiate from the mouth when females are interacting with males.

As predicted, we also found that male and female bleats produced during copulation had higher mean F0 than those produced during the pre-copulatory stage. Female bark mean F0 was also higher during the pre-copulatory phase of successful breeding introductions than it was during unsuccessful introductions. These findings are likely to indicate increased sexual arousal [25,33,34], and are also consistent with Morton's motivation-structural rules [47], in which higher pitched sounds are expected in friendly/appeasing contexts. We suggest that female appeasement of males prior to and during copulation is a possibility because giant pandas are a solitary-living species that must overcome aggressive tendencies towards conspecifics for mating to occur. Moreover, male and female giant pandas can be aggressive towards one another during mating attempts [31] and mutual appeasement of sexual partners may go some way to avoiding this. The production of higher F0 moans prior to copulation may also indicate that appeasement of males is more important just prior to copulation than it is once copulation has been initiated.

Contrary to our predictions, the pre-copulatory phase of successful introductions was characterized by relatively fewer female chirps than unsuccessful introductions. Chirps produced during unsuccessful breeding introductions also had higher F0 and a greater prevalence of nonlinear phenomena. According to Morton's motivation-structural rules [47], the increased F0 of chirps produced during unsuccessful breeding introductions could reflect an effort to appease a male showing incipient signs of aggression in breeding introductions that ultimately failed. Alternatively, increased F0 could reflect an attempt to produce a more conspicuous signal to draw attention to the caller, perhaps to encourage male sexual attention. In addition, a higher incidence of nonlinear phenomena in female chirps is likely to increase the auditory impact of these calls [48,49] and recent studies of mammal vocal communication have shown that vocalizations with nonlinear phenomena are particularly evocative and hard to ignore [50–52]. Accordingly, we suggest that the relatively high F0 and greater incidence of nonlinear phenomena in chirps produced during unsuccessful breeding introductions reflects an attempt by females to grab and maintain the attention of male receivers that may be reluctant to mate.

Barks and moans produced during unsuccessful introductions also contained more nonlinear phenomena than those produced during the pre-copulatory stage of successful breeding introductions. In addition, and contrary to our predictions, moans and barks produced during copulation had less nonlinear phenomena than those produced prior to copulation. Taken together, these results provide further support for the contention that nonlinear phenomena in female giant panda vocalizations functions to elicit attention from male receivers prior to copulation. It is, therefore, surprising that chirps produced during copulation had more nonlinear phenomena than those given during the pre-copulatory phase of successful breeding introductions, when it is assumed that male attention had already been gained. We suggest that the production of chirps with increased nonlinear phenomena during copulation could conceivably help to regain male attention when mounting has been interrupted, which often happens during giant panda mating attempts [31]. Future playback studies are now required to examine the response of male giant pandas to female chirps, barks and moans with and without nonlinear phenomena, to conclusively demonstrate whether this pervasive feature of mammal vocalizations functions to attract and maintain the attention of conspecific receivers in the giant panda's vocal communication system.

Relatively few female barks and no roars were observed during the pre-copulatory phase of successful introductions. Female barks were also longer in duration during unsuccessful breeding introductions than the pre-copulatory phase of successful introductions. These findings confirm that the production of barks and roars is not conducive for promoting contact and/or sexual motivation prior to copulation, and are in accordance with these vocalizations' presumed role as an aggressive threat to receivers [12,53]. Interestingly, we also found that female roars were only produced during the copulation phase of successful breeding introductions, and barks produced during successful breeding introductions were significantly longer in duration once copulation had begun. Roars and barks are thought to reflect aggression [12], which would obviously not be compatible with a successful mating. However, observations of breeding introductions confirm that it is common for the female giant pandas to be aggressive immediately after copulation, expressed as breaking away from the male, barking, roaring and lunging [32]. The roars and barks that we observed during the copulation phase of successful breeding introductions are, therefore, likely to reflect female intolerance of close contact once copulation draws to a close.

A recent emphasis for giant panda breeding programmes has been to promote natural mating [32,41,42,54] and knowledge about reproductive behaviour is at the heart of this endeavour. We suggest that our findings could add to a growing body of research now informing captive breeding efforts. For instance, animal caretakers could be trained to recognize vocal behaviour that predicts successful copulation versus vocalizations indicating likely failure, which may aid managers in decisions about continuing or terminating mating introductions. Toward this end, we have provided exemplar calls in this study's electronic supplementary material to be used as a training tool. Examples of important distinctions for managers to make include calls with and without nonlinear phenomena. A high occurrence of barks with nonlinear phenomena (electronic supplementary material, audio S1.wav) would indicate that the mating attempt should most probably be aborted, as would the production of any roars (electronic supplementary material, audio S2.wav). Chirps with nonlinear phenomena (electronic supplementary material, audio S3.wav and audio S4.wav), if our interpretation is correct, may indicate that the mating introduction is at risk of failure, that the female perceives this risk, and that she is making efforts to appease and sexually motivate the male, and thus allowing more time for the mating introduction to succeed may be warranted. Conversely, long duration male bleats (electronic supplementary material, audio S5.wav) and more tonal female moans and barks (electronic supplementary material, audio S6.wav and audio S7.wav) would be predictive of behavioural compatibility and subsequent copulation. A shift to higher F0 bleats would also be indicative of male intromission and the start of copulation (electronic supplementary material, audio S8.wav). Previous studies have shown that giant pandas display mating preferences [41,42] and use multimodal signalling in the lead up to mating [6]. The findings of the current study illustrate that vocal exchanges are crucial for signalling motivational state and intention to mate once close contact between male and female has been made. Whether giant pandas base mate choice decisions on vocal characteristics, and how this information interacts with other signalling modalities, are key questions for future research.

## Supplementary Material

Descriptions of Electronic Supplementary Material.docx

## Supplementary Material

Dataset.sav

## Supplementary Material

Audio S1.wav

## Supplementary Material

Audio S2.wav

## Supplementary Material

Audio S3.wav

## Supplementary Material

Audio S4.wav

## Supplementary Material

Audio S5.wav

## Supplementary Material

Audio S6.wav

## Supplementary Material

Audio S7.wav

## Supplementary Material

Audio S8.wav

## Data accessibility

The data used in this study are available as electronic supplementary material.
