# Supplementary material for: Vocal behaviour predicts mating success in giant pandas
Source: R Soc Open Sci. 2018 Oct 31;5(10):181323. doi: 10.1098/rsos.181323 (PMC6227945; doi:10.1098/rsos.181323)
Supplement: Descriptions of Electronic Supplementary Material.docx [file rsos181323supp1.docx]

**Electronic Supplementary Material**

**Dataset.sav:** Acoustic data for the different giant panda vocalisations produced during the breeding introductions.

**Audio S1.wav:** Female giant panda bark with deterministic chaos. Deterministic chaos consists of episodes of non-random broadband noise, and confers a harsh sounding quality to the call.

**Audio S2.wav:** Female giant panda roar. Roars always contain deterministic chaos.

**Audio S3.wav:**  Female giant panda chirp with a frequency jump. Frequency jumps are abrupt discontinuous changes in F0.

**Audio S4.wav:** Female giant panda chirp with biphonantion. Biphonantion is the occurrence of two simultaneous but independent F0s.

**Audio S5.wav:** Male giant panda bleat. Bleats are characterised by rapid F0 modulation.

**Audio S6.wav:** Tonal female giant panda moan. Tonal moans do not contain non-linear phenomena.

**Audio S7.wav:** Tonal female giant panda bark. Tonal barks do not contain non-linear phenomena.

**Audio S8.wav:** Male giant panda bleat delivered during copulation. Bleats produced during copulation have significantly higher F0 than those produced prior to copulation.
